# Supplementary material for: Survey for Major Grapevine Viruses in Commercial Vineyards of Northwestern Argentina
Source: Plants (Basel). 2022 Jun 28;11(13):1720. doi: 10.3390/plants11131720 (PMC9268931; doi:10.3390/plants11131720)
Supplement: Supplementary file 1 [file plants-11-01720-s001.zip › plants-1697171-supplementary.pdf]

Table S1. Details of the screening of eight grapevine viruses analyzed in a total of 103 mature cane samples collected from randomly selected vineyard blocks.

N°	Province[a]	Locality	Variety	Age	Screened Virus[b]							
				(years)	GLRaV-1	GLRaV-2	GLRaV-3	GLRaV-4	GRBaV	GFLV	GRSPaV	GVA
1	SA	Cachi	Malbec	12	-	-	-	-	-	1	-	-
2	SA	Cachi	Tannat	12	-	-	1	-	-	1	-	-
3	SA	Cachi	Malbec	12	-	-	-	-	-	-	-	-
4	SA	Cachi	Criolla tinta	14	-	-	-	-	-	1	-	-
5	SA	Cachi	Malbec	9	-	-	-	-	-	-	-	-
6	SA	Cachi	Malbec	15	-	-	1	-	-	-	-	-
7	SA	Cachi	Merlot	14	-	-	1	-	-	1	-	-
8	SA	Cachi	Malbec	14	-	-	-	-	-	-	-	-
9	SA	Cachi	Malbec	7	1	-	-	-	-	-	-	1
10	SA	Cachi	Malbec	5	-	1	1	-	-	1	-	1
11	SA	Cachi	Aspirant	3	-	-	-	-	-	-	-	1
12	SA	Cachi	San Giovese	18	-	-	-	-	-	-	-	-
13	SA	Cachi	Torrontes	12	-	-	-	-	-	-	-	-
14	SA	Cachi	Merlot	19	-	-	-	-	-	-	-	-
15	SA	Cachi	Tannat	19	-	-	-	1	-	-	-	1
16	SA	Cachi	Cabernet Sauvignon	19	-	-	-	-	-	-	-	-
17	SA	Cachi	Malbec	18	1	-	-	-	-	-	-	-
18	SA	Cachi	Petit Verdot	19	1	-	-	-	-	-	-	-
19	SA	Cachi	Criolla tinta	27	-	-	1	-	-	-	-	1
20	SA	Cachi	Malbec	26	-	-	-	-	-	-	-	-
21	SA	Cachi	Malbec	27	-	-	-	-	-	-	-	-
22	SA	Cachi	Merlot	27	-	1	-	-	-	-	-	-
23	SA	Cachi	Malbec	10	-	1	-	-	-	-	-	-
24	SA	Cachi	Malbec	10	-	-	1	-	-	-	-	1
25	SA	Cachi	Criolla Cereza	24	-	1	-	-	-	-	-	-
26	SA	Cachi	Cabernet Sauvignon	21	-	-	-	-	-	-	-	-
27	SA	Cachi	Malbec	5	-	-	-	-	-	-	-	-
28	SA	Cafayate	Cabernet Sauvignon	23	-	-	1	-	-	1	-	1
29	SA	Cafayate	Malbec	10	-	-	1	-	-	-	-	-
30	SA	Cafayate	Malbec	23	-	-	1	-	-	1	1	-
31	SA	Cafayate	Malbec	16	-	-	1	-	-	1	-	-
32	SA	Cafayate	Malbec	16	-	-	1	-	-	-	-	-
33	SA	Cafayate	Malbec	16	1	-	1	-	-	-	-	-
34	SA	Cafayate	Cabernet Sauvignon	37	-	-	1	-	-	-	-	-
35	SA	Cafayate	Cabernet Franc	3	1	-	1	-	-	-	-	1

36	SA	Cafayate	Cabernet Franc	3	-	1	1	-	-	-	-	1
37	SA	Cafayate	tanat	25	-	-	1	-	-	-	-	-
38	SA	Cafayate	tanat	25	1	-	1	-	-	-	-	-
39	SA	Cafayate	tanat	25	1	-	1	-	-	1	-	-
40	SA	Cafayate	Malbec	12	1	-	1	-	-	1	-	1
41	SA	Cafayate	Malbec	12	-	-	1	-	-	-	-	-
42	SA	Cafayate	Malbec	12	-	-	1	-	-	-	-	-
43	SA	Cafayate	Malbec	12	1	-	1	-	-	-	-	-
44	SA	Cafayate	Malbec	12	-	-	1	-	-	-	-	-
45	SA	Cafayate	Malbec	12	-	-	1	-	-	-	-	-
46	SA	Cafayate	Malbec	12	-	-	1	-	-	-	-	-
47	SA	Cafayate	Malbec	13	-	-	1	-	-	-	1	-
48	SA	Cafayate	Malbec	13	-	-	1	-	-	-	-	-
49	SA	Cafayate	Malbec	13	-	-	1	-	-	1	-	-
50	SA	Molinos	Cabernet Sauvignon	5	-	-	-	-	-	-	-	-
51	SA	Molinos	Tannat	15	-	-	1	-	-	-	-	1
52	SA	Molinos	Tannat	15	-	-	-	-	-	-	-	-
53	SA	Molinos	Malbec	10	-	1	-	-	-	-	-	-
54	SA	Molinos	Malbec	10	-	1	-	-	-	1	-	-
55	SA	Molinos	Malbec	10	-	-	-	-	-	-	-	-
56	SA	Molinos	Tannat	10	1	-	-	-	-	-	-	-
57	SA	Molinos	Malbec	10	-	-	-	-	-	-	1	-
58	SA	Molinos	Syrah	10	-	-	-	-	-	-	-	-
59	SA	Molinos	Malbec	10	-	-	-	-	-	-	-	-
60	SA	Molinos	Petit Verdot	10	-	-	-	-	-	-	-	-
61	SAL	Molinos	Malbec	19	-	-	-	-	-	-	-	-
62	SAL	Molinos	Cabernet Sauvignon	19	-	-	-	-	-	-	-	-
63	SAL	Molinos	Cabernet Sauvignon	19	-	-	-	-	-	-	-	-
64	SA	Molinos	Malbec	15	-	-	-	-	-	-	-	-
65	SA	Molinos	Malbec	15	-	-	-	1	-	-	-	-
66	SA	Molinos	Malbec	15	-	-	-	-	-	-	-	-
67	SA	Molinos	Malbec	13	-	-	-	-	-	-	-	-
68	SA	Molinos	Malbec	13	-	-	-	-	-	-	-	-
69	SA	Molinos	Malbec	13	-	-	-	-	-	1	-	-
70	SA	Molinos	Malbec	13	-	-	-	-	-	-	-	-
71	SA	Molinos	Malbec	13	-	-	-	-	-	1	-	-
72	SA	Molinos	Cabernet Sauvignon	21	-	-	-	-	-	-	-	-
73	SA	Molinos	Cabernet Sauvignon	22	-	-	-	-	-	-	-	-
74	SA	Molinos	Malbec	11	-	-	-	-	-	-	-	-
75	SA	Molinos	Malbec	11	-	-	-	-	-	1	-	-
76	SA	Molinos	Malbec	10	-	-	-	-	-	-	-	-

77	SA	San Carlos	Malbec	12	-	-	-	-	-	-	-	-
78	SA	San Carlos	Malbec	12	-	-	1	-	-	-	-	1
79	SA	San Carlos	Malbec	12	-	-	1	1	-	-	-	1
80	SAL	San Carlos	Cabernet Sauvignon	12	-	-	1	-	-	-	-	-
81	SAL	San Carlos	Malbec	12	-	-	-	-	-	1	-	-
82	TUC	Tafi del valle	Malbec	13	-	-	-	-	-	1	-	-
83	TUC	Tafi del valle	Malbec	13	-	-	-	-	-	1	-	-
84	TUC	Tafi del valle	Malbec	13	-	-	-	-	-	1	-	-
85	TUC	Tafi del valle	Malbec	13	-	1	-	-	-	-	-	-
86	TUC	Tafi del valle	Malbec	13	-	-	-	-	-	-	-	-
87	TUC	Tafi del valle	Malbec	4	-	-	-	-	-	-	-	-
88	TUC	Tafi del valle	Malbec	12	-	-	-	-	-	-	-	-
89	TUC	Tafi del valle	Malbec	12	-	-	1	-	-	-	-	-
90	TUC	Tafi del valle	Malbec	6	-	-	-	-	-	-	-	-
91	TUC	Tafi del valle	Criolla Cereza	7	-	-	-	-	-	1	-	-
92	TUC	Tafi del valle	Malbec	12	-	-	1	-	-	-	-	-
93	TUC	Tafi del valle	Malbec	12	-	-	-	-	-	1	-	-
94	TUC	Tafi del valle	Malbec	12	-	-	1	-	-	-	-	-
95	TUC	Tafi del valle	Malbec	12	-	-	-	-	-	-	-	-
96	TUC	Tafi del valle	Malbec	12	-	-	-	-	-	-	-	-
97	TUC	Tafi del valle	Malbec	12	-	-	-	-	-			
